# Diagnostic Re-Evaluation and Potential Predictor Factors of Transient and Permanent Congenital Hypothyroidism in Eutopic Thyroid Gland

**DOI:** 10.3390/jcm10235583

**Published:** 2021-11-27

**Authors:** Gerdi Tuli, Jessica Munarin, Luisa De Sanctis

**Affiliations:** 1 Department of Pediatric Endocrinology, Regina Margherita Children’s Hospital, City of Health and Science University Hospital of Turin, 10126 Turin, Italy; jessica.munarin@edu.unito.it (J.M.); luisa.desanctis@unito.it (L.D.S.); 2 Department of Public Health and Pediatric Sciences, University of Turin, 10124 Turin, Italy

**Keywords:** transient congenital hypothyroidism, permanent congenital hypothyroidism, eutopic thyroid gland, predictive factors

## Abstract

Background: The incidence of congenital hypothyroidism (CH) has increased over the years, and many predictors for detecting newborns with transient forms (TCH) as early as possible have been considered. Methods: All newborns diagnosed with primary CH and eutopic gland in the Piedmont region of Italy in the period of January 2014–June 2019 were enrolled and re-evaluated at the age of 2 years. Results: 105 newborns were diagnosed with CH during the study period. Dyshormonogenesis was observed in 55/105. At re-evaluation, we found that 52.7% had permanent CH (PCH), while 47.3% had TCH. Male/female rate, TSH levels at diagnosis, levothyroxine requirement at withdrawal and extra-thyroid congenital malformations rate were higher in the PCH group (*p* = 0.02, *p* = 0.009, *p* = 0.02 and *p* = 0.01), while fT4 levels at diagnosis were lower (*p* = 0.03). Sensitivity of 72.4% and specificity of 80.7% for serum TSH above 60 mcUI/mL, sensitivity of 73% and specificity of 72.4% for serum fT4 level below 7.2 pg/mL and sensitivity of 66% and specificity of 68% for drug requirement above 2.25 mcg/kg/day were observed in PCH. Conclusions: Demographic, clinical and hormonal data at diagnosis and levothyroxine requirement during the first two years should be adequately monitored to identify infants who are most likely to discontinue therapy after the age of 24 months.

## 1. Introduction

Congenital hypothyroidism (CH) is the most common congenital endocrine disorder, with classically reported incidence of 1:3.000–1:4.000 births [1]. In recent years, an increasing incidence has been reported, and it is influenced by many factors, e.g., the cutoff of Thyroid Stimulating Hormone (TSH) at neonatal screening on a dry blood spot (DBS), as it has been progressively lowered in almost all countries. In addition, another important fact is the increase in the population of newborns at risk of developing CH, such as preterm babies or twins. Other factors that can influence the increased incidence include continuous ethnic population change, iodine deficiency during pregnancy, the presence of CH-associated syndromes and genetic mutations in genes responsible for thyroid ontogenesis or function. Despite the presence of Consensus Guidelines for the management of CH in pediatric age, the screening strategy and management of CH, especially where TSH elevation is mild, differ significantly between individual centers [2].

After reporting by the national screening center, the diagnosis of CH should be confirmed by evaluating the thyroid hormonal profile of TSH, free Thyroxine (fT4), free triiodothyronine (fT3), thyroglobulin and radiological evaluation by ^99^Tc or ^121^I scintiscan and/or ultrasound. Positive titer of anti-peroxidase (AbTPO), anti-thyroglobulin (AbHTG) and anti-TSH receptor antibodies should also always be omitted out at the time of diagnosis. 

Within CH, thyroid dysgenesis, including total agenesis, ectopy or hypoplasia/hemiagenesis, has been described as the most frequent form, responsible for 75–85% of cases, while thyroid dyshormonogenesis has been classically reported in 15–25%. In recent years, the percentages have changed significantly, with the diagnostic category of eutopic gland reported in up to 40–50% of cases [1,2].

Diagnostic re-evaluation is therefore very important in all patients diagnosed with CH and eutopic thyroid gland detected by scintiscan or ultrasound to differentiate permanent CH (PCH) from transient CH (TCH) forms. Discontinuation of levothyroxine therapy should be performed at the age of 2 to 3 years, for a period of 4 weeks. TSH levels <10 mcUI/mL and normal fT4 levels are therefore indicative of TCH. Current data indicate TCH in 20–89% of cases of children with eutopic gland [3,4,5,6,7,8,9,10,11,12,13,14,15,16]. The most important predictive factors of TCH appear to be TSH and fT4 levels at diagnosis and levothyroxine requirement in the first two years of life, but also gender, delivery, prematurity, low birth weight (LBW), maternal risk factors, family history of thyroid disease and consanguinity have been considered as risk factors [17,18,19,20,21,22,23,24,25,26,27,28,29,30]. 

The aim of this study was to determine the rate of TCH in infants diagnosed with CH with eutopic thyroid in the Italian Region of Piedmont and to analyze the potential predictors of TCH in early infancy.

## 2. Materials and Methods

All newborns diagnosed with primary CH at the neonatal screening program in the Piedmont region in Italy in the period January 2014–June 2019 were enrolled. All TSH detection tests on dried blood spot (DBS), measured as serum-equivalents after multiplying by 2.2, were performed at the regional reference center for Neonatal Screening at Regina Margherita Children’s Hospital. 

Infants with syndromic CH or chromosomal abnormalities, neonatal TCH or isolated hyperthyrotropinemia in which replacement treatment was not initiated were excluded. Confirmation of the diagnosis of CH was based on blood levels of TSH, fT4, fT3 and thyroglobulin, and on radiological evaluation with ^99^Tc scintigraphy and ultrasound examination in all cases of suspected thyroid agenesis. AbTPO and AbHTG antibodies were evaluated in the case of unknown or positive maternal antibodies titer. All blood tests were performed in a single laboratory. 

Diagnostic re-evaluation was performed at the age of 2 years by prior suspension of levothyroxine for 4 weeks. Subsequent hormonal assessment included TSH and fT4 evaluation. A TCH was defined when the TSH level was <10 mcUI/mL and fT4 was within the normal range for a period of at least one year.

Statistical analyses and graphs were performed through GraphPad 7 software (GraphPad Software, La Jolla, CA, USA), using the chi-squared test and multivariate analysis through logistic regression to compare differences between groups. Receiver Operating Characteristic (ROC) curves were analyzed for serum TSH and fT4 levels at diagnosis and levothyroxine requirement at withdrawal, as well as for sensitivity and specificity. 

## 3. Results

In the period 2014–2019, in the Italian region of Piedmont, 105 newborns (57 males and 48 females) were diagnosed with CH (incidence of 1:1090) [1]. 

Thyroid scintigraphy revealed gland dysgenesis in 47.6% (50/105) (thyroid agenesis in 14.3% (15/105) of cases, and ectopia and thyroid hypoplasia in 23.8% (25/105) and 9.5% (10/105) respectively); a defect in hormonogenesis with eutopic gland was observed in 52.4% (55/105) of the cases. None of the subjects with agenesis or ectopia at scintiscan was found to have eutopic thyroid on ultrasound evaluation. All subjects with thyroid in situ were re-evaluated at a mean age of 2.4 years, and 29/55 (52.7%) were shown to have PCH, while 26/55 (47.3%) had a TCH. Demographic, clinical and hormonal data of subjects with PCH and TCH are represented in Table 1. 

Considering gestational age, delivery at birth, family history of thyroid disease, neonatal weight and length, fT3 levels, starting dose of levothyroxine and levothyroxine requirement during the first and the second year of life, no difference was observed between the two groups. The male/female rate was significantly higher in the PCH group (M/F = 3.1 vs. 0.86, *p* = 0.02). TSH values were significantly higher in PCH group (197.3 ± 44.5 μUI/mL) than in the TCH group (55.01 ± 7.33) (*p* = 0.009), while fT4 levels were lower in subjects with PCH (6.92 ± 0.72 vs. 9.29 ± 1.98 pg/mL, *p* = 0.03). Of the 26 subjects with TCH, 22/26 had normal TSH levels after treatment withdrawal, while 4/26 had TSH levels ranging from 5.6 to 7.9 mcUI/mL (considering upper normal range 5.5 mcUI/mL) during a mean follow-up period of 3.5 years. AbTPO and AbHTG resulted in being negative in all subjects included in the study. The requirement for levothyroxine at the time of withdrawal was higher in the PCH group (2.69 ± 0.14 vs. 2.14 ± 0.19) (*p* = 0.02). TSH levels after treatment withdrawal were higher in the subjects with PCH (23.37 ± 13.6 vs. 4.1 ± 0.31, *p* = 0.007), while fT4 levels were lower 9.54 ± 1.22 vs. 13.13 ± 0.33, *p* = 0.004). Extra-thyroid congenital malformations were observed in 15 subjects with PCH (51.7%) and five subjects with TCH (19.2%) (*p* = 0.01). The most common malformations in the PCH group involved cardiac system (62.5%, 5/8), urogenital tract (75%, 3/4), gastrointestinal tract (100%, 5/5) and musculoskeletal system (66.7%, 2/3). 

**Figure 1:**
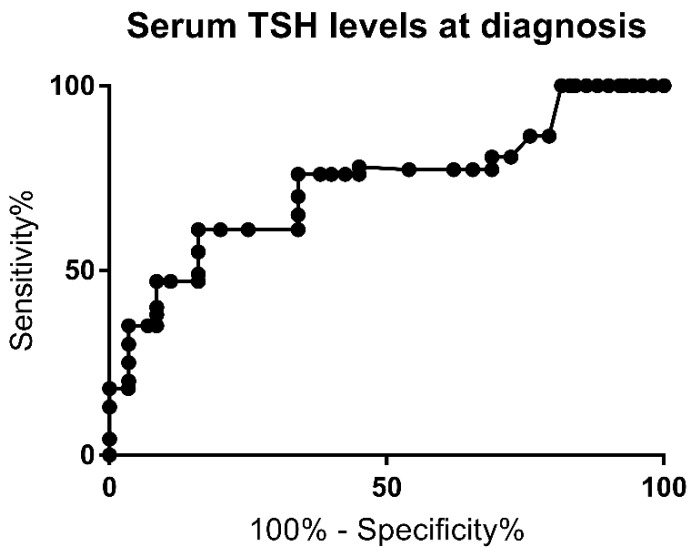
Receiver Operating Curve (ROC) for serum TSH level at the time of diagnosis.

The ROC curve for TSH serum levels at diagnosis is represented in Figure 1. The area under the curve (AUC) was 0.8 (*p* = 0.0002). 

A serum TSH level above 60 mcUI/mL was found to have a sensitivity of 72.4% and a specificity of 80.7% for PCH (likelihood ratio of 2.8). Multivariate analysis for this cutoff also showed a higher risk for PCH (*p* = 0.001) and was associated with a higher risk of extra-thyroid malformation rate (*p* = 0.001). The highest specificity (100%) was achieved for TSH levels below 21.9 mcUI/L.

**Figure 2:**
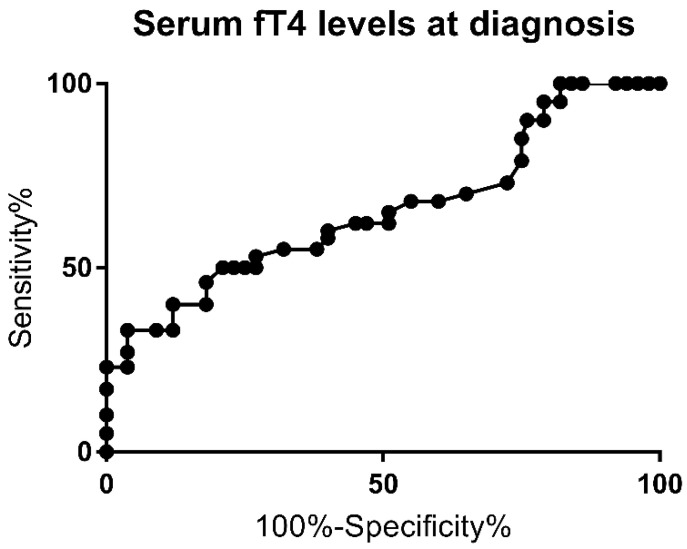
Receiver Operating Curve (ROC) for fT4 level at the time of diagnosis.

The ROC curve for fT4 levels at diagnosis is shown in Figure 2. The AUC was 0.72 (*p* = 0.006). 

A 73% sensitivity and 72.4% specificity for PCH (likelihood ratio of 1.86) were observed for serum fT4 levels below 7.2 pg/mL. The highest specificity (100%) was achieved for fT4 levels above 13.1 pg/mL. Multivariate analysis for this cutoff showed a higher risk for PCH (*p* = 0.0005), as well, and this was associated with a higher risk of extra-thyroid malformation rate (*p* = 0.0004). 

**Figure 3:**
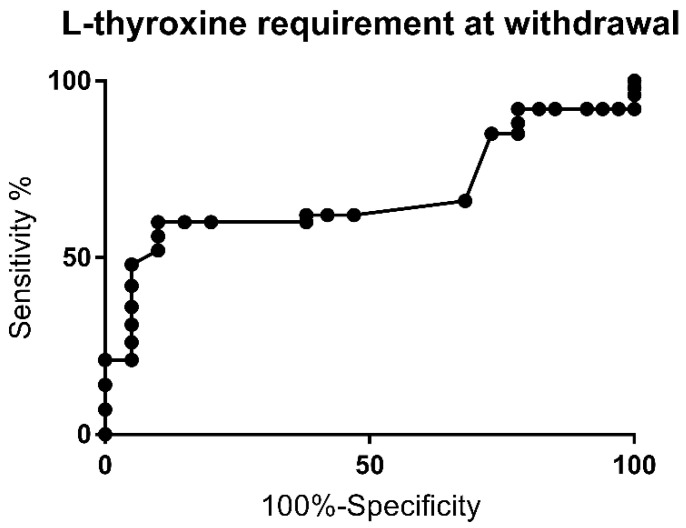
Receiver Operating Curve (ROC) for levothyroxine requirement upon withdrawal of therapy.

The ROC curve for levothyroxine requirement at the time of withdrawal is shown in Figure 3. The AUC was 0.7 (*p* = 0.02). 

A drug requirement above 2.25 mcg/kg/day was observed to have a sensitivity of 66% and specificity of 68% for PCH (likelihood ratio of 2.1). The highest specificity (100%) was achieved with doses above 3.5, 3.73 and 4.18 mcg/kg/day, respectively. Multivariate analysis for this cutoff showed a higher risk for PCH (*p* = 0.002) and was associated with a higher risk of extra-thyroid malformation rate (*p* = 0.002).

In the multiple logistic model, PCH remained significantly associated with TSH above 60 mcUI/mL (*p* < 0.0001), fT4 levels inferior to 7.2 pg/mL (*p* < 0.002) at diagnosis, associated malformations (*p* = 0.005), male gender (*p* = 0.02) and levothyroxine requirement above 2.25 mcg/kg/day at treatment-withdrawal (*p* < 0.0005) requirement in the first two years.

## 4. Discussion

The incidence of CH has increased in recent years in nearly all countries that have a national newborn screening program. The latest update of the European Society of Pediatric Endocrinology (ESPE) and the ENDO-European Reference Network (ENDO-ERN) Consensus Guidelines for CH reports an incidence of 1:2000–3000, while the incidence in the Italian Region of Piedmont was 1:1090 births in the period 2014–2019 [1,2]. The continuously lowered cutoff of TSH detection on dry blood spots (DBSs) is the most important factor influencing the increase in incidence. This strategy has allowed the diagnosis of even mild forms of CH, particularly those with eutopic thyroid, which currently include 40–50% of CH [2]. Environmental, genetic and ethnic factors also have important consequences on the increase in incidence [1,2]. 

At the age of two to three years, all infants with CH and eutopic gland should be referred for discontinuation of therapy and reconsideration of diagnosis. There are two strategies for withdrawal: immediate discontinuation of treatment for 4 weeks or gradual dose reduction until the treatment is discontinued. levothyroxine treatment is then promptly resumed if TSH levels are above 10 mcUI/mL and/or fT4 levels are below the normal range for age. The rate of TCH in infants with eutopic thyroid gland in the main studies differs from 20 to 89%, as represented in Table 2 [3,4,5,6,7,8,9,10,11,12,13,14,15,16]. 

This variability can be explained by many factors, such as the inclusion/exclusion criteria, the cutoff of TSH at DBS for diagnosis or the recall strategy, the age of discontinuation of therapy. If the condition of CH due to ectopic gland or athyreosis is also considered, this rate is reduced to 6.7–60% [17,18,19,20,21,22,23,24,25,26,27,28,29,30]. In our current study, the TCH rate was 47.3% when considering only subjects with eutopic thyroid, and 24.8% when considering the whole cohort. The withdrawal timing in our cohort was at a mean age of 2.4 years, slightly earlier than the recommended age of 3 years. Normal thyroid hormones profile and constant levothyroxine requirement, rather than age, were considered for withdrawal timing.

Many authors have analyzed the risk factors for developing PCH by finding female gender, higher TSH and lower fT4 levels at diagnosis and higher levothyroxine requirement as predictors [3,4,5,6,7,8,9,10,11,12,13,14,15,16,17,18,19,20,21,22,23,24,25,26,27,28,29,30]. Our data confirm that the thyroid hormonal profile at diagnosis and the levothyroxine requirement upon discontinuation of treatment are predictive for the permanent condition, as well as the presence of extra-thyroid malformations. To our knowledge, the association between extra-thyroid malformations and PCH has not yet been reported in the literature. Contrary to other reports [4,5,7,8,9,10,11,12,13,15,16,28,29], we found male sex to be significantly related to PCH. 

We found that a serum TSH level above 60 mcUI/mL has a sensitivity of 72.4% and a specificity of 80.7% for PCH, with a likelihood ratio of 2.8 and AUC of 0.8. Multivariate analysis for this cutoff also showed a higher risk for PCH and was also associated with a higher risk of extra-thyroid malformation rate. Oron et al. [11] and Kang [8] reported a similar cutoff for serum TSH levels at diagnosis, while other studies reported a lower cutoff for TSH, between 28.4 and 43.35 mcUI/mL [8,11,31,32]. In our cohort, specificity of 100% was achieved for TSH levels below 21.9 mcUI/mL. 

Furthermore, in the present study, a serum fT4 level below 7.2 pg/mL was found to have a sensitivity of 73% and a specificity of 72.4% for PCH and a likelihood ratio of 1.86, while the AUC was 0.72. The multivariate analysis for this cutoff also showed a higher risk for PCH and was also associated with a higher risk of extra-thyroid malformation rate. 

A drug requirement above 2.25 mcg/kg/day was observed to have a sensitivity of 66% and sensibility of 68%, likelihood ratio of 2.1 and AUC of 0.7 for PCH development. A similar cutoff for levothyroxine requirement at 24 months was found by other authors, while Messina et al. reported a levothyroxine dose greater than 1.45 mcg/kg/day at 24 months as predictive for PCH [11,16,18]. Other authors have reported that higher doses of levothyroxine at the time of withdrawal, of 2.86, 3.25 and 4.1 mcg/kg/day, respectively, suggest a PCH [5,8,13]. In this study, a specificity of 100% was found for the dose of levothyroxine above 3.5 mcg/kg/day. The high variability of levothyroxine requirement upon discontinuation of treatment is mainly related to the age of withdrawal and the diagnosis criteria at birth. In the present study, there was no difference in drug demand during the first two years of follow-up between subjects with permanent and transient forms. Other authors who have found significant differences report lower AUCs than the data at 24 months; therefore, the diagnostic reconsideration should always be performed after the age of two, considering the sequelae that the discontinuation could have on neuropsychological development [7].

The limitations of this study are related to the collection of retrospective data and the different management of CH compared to other studies with which our current data were compared. The variability in diagnostic criteria and further management of CH, including the age of withdrawal of therapy at the time of reconsideration, differ consistently between individual centers.

## 5. Conclusions

CH is the most common congenital endocrine disorder, with an increasing incidence in recent years. To better define each condition, radiological evaluation at diagnosis should include both scintiscan and ultrasound. Demographic, clinical, hormonal and radiological data at diagnosis and levothyroxine requirement during the first two years of follow-up should be adequately monitored to identify infants who are most likely to discontinue therapy after the age of 24 months.

However, the current data do not allow for clarification of the natural course of transient hypothyroidism in early infancy and do not indicate whether subjects with TCH need treatment with levothyroxine later in the evolutive age; therefore, long-term follow-up is required in this population.

## Figures and Tables

**Table 1:** Demographic, clinical and hormonal data of the 55 infants affected by CH and eutopic thyroid gland.

		Permanent CH (PCH) 29 Subjects = 52.7%	Transient CH (TCH) 26 Subjects = 47.3%	*p*
Gender	Male	22	12	**0.02**
	Female	7	14	
Gestational Age	At term	21	17	0.6
	Preterm	8	9	
Delivery	Vaginal	15	11	0.5
	Caesarean section	14	15	
Family history for thyroid disease		7	10	0.3
Neonatal Weight (g)		2285 ± 156.7	2602 ± 129.3	0.1
Neonatal Length (cm)		47.7 ± 0.76	46.3 ± 1.05	0.3
Blood TSH mcUI/mL		197.3 ± 44.5	55.01 ± 7.33	**0.009**
FT4 pg/mL		6.92 ± 0.72	9.29 ± 1.98	**0.03**
FT3 pg/mL		3.75 ± 0.25	4.19 ± 0.48	0.3
Levothyroxine initial dose		8.37 ± 0.43	9.13 ± 0.44	0.23
Levothyroxine requirement in the 1st year		5.12 ± 0.36	5.61 ± 0.55	0.46
Levothyroxine requirement in the 2nd year		2.77 ± 0.19	2.74 ± 0.2	0.9
Levothyroxine requirement at withdrawal		2.69 ± 0.14	2.14 ± 0.19	**0.02**
TSH mcUI/mL prior to withdrawal		2.81 ± 0.74	1.98 ± 0.27	0.2
FT4 pg/mL prior to withdrawal		14.53 ± 0.56	14.45 ± 0.44	0.9
TSH mcUI/mL after withdrawal		23.37 ± 13.6	4.1 ± 0.31	**0.007**
FT4 pg/mL after withdrawal		9.54 ± 1.22	13.13 ± 0.33	**0.004**
Malformations		15	5	**0.01**

**Table 2:** Main studies on TCH and PCH in infants with CH and eutopic thyroid gland.

Study	Year	No. of Subjects with Eutopic Thyroid	TCH Rate	Main Findings
Nair et al. [3]	2010	23	80%	Significant higher TSH in PCH subjects
Rabbiosi et al. [4]	2013	84	67%	Same clinical outcome in patients with screening TSH values < 20 and >20 mcUI/mL. M/F ratio: 0.88 for TCH vs. 1.23 for PCH (not significant). Malformations rate 12.5% for TCH vs. 13.7% for PCH.
Cho et al. [5]	2014	56	45%	TSH at diagnosis and levothyroxine requirement found to be predictive. M/F ratio: 0.92 for TCH vs. 0.93 for PCH.
Messina et al. [6]	2015	64	72%	TSH at diagnosis and levothyroxine requirement found to be predictive.
Kara et al. [7]	2016	86	73%	Levothyroxine requirement found to be predictive. M/F ratio: 1.7 for TCH vs. 0.7 for PCH.
Kang et al. [8]	2017	20	50%	TSH at diagnosis and levothyroxine requirement found to be predictive. M/F ratio: 1.3 for TCH vs. 0.6 for PCH.
Park et al. [9]	2017	100	65%	TSH at diagnosis and levothyroxine requirement. M/F ratio: 1.2 for TCH vs. 1.1 for PCH found to be predictive.
Saba et al. [10]	2018	92	54%	Levothyroxine requirement found to be predictive. M/F ratio: 1.1 for TCH vs. 1.05 for PCH.
Oron et al. [11]	2018	84	20%	Levothyroxine requirement found to be predictive. M/F ratio: 1.43 for TCH vs. 0.86 for PCH.
Higuchi et al. [12]	2019	30	50%	Levothyroxine requirement found to be predictive. M/F ratio: 0.9 for TCH vs. 1.4 for PCH (not significant).
Park et al. [13]	2019	80	89%	Levothyroxine requirement found to be predictive. M/F ratio: 0.97 for TCH vs. 0.55 for PCH. Malformations rate not reported.
Asena et al. [14]	2020	186	29%	Levothyroxine requirement found to be predictive.
Long et al. [15]	2020	190	45%	Mutations related to thyroid dysgenesis are more likely to have PCH. M/F ratio: 1.09 for TCH vs. 1.1 for PCH. (not significant).
Chen et al. [16]	2021	508	66%	Levothyroxine requirement and familial history of CH found to be predictive. M/F ratio: 1.22 for TCH vs. 1.58 for PCH (not significant).
Current study	2021	55	47%	M/F ratio: 0.86 for TCH vs. 3.1 for PCH. TSH above 60 mcUI/mL and fT4 inferior to 7.2 pg/mL at diagnosis and levothyroxine requirement above 2.25 mcg/kg/day found to be predictive of PCH.

## Data Availability

All data present in the study are available upon request to the corresponding author.

## References

[1] Tuli G., Munarin J., Tessaris D., Matarazzo P., Einaudi S., De Sanctis L. (2021). Incidence of primary congenital hypothyroidism and relationship between diagnostic categories and associated malformations. Endocrine.

[2] van Trotsenburg P., Stoupa A., Léger J., Rohrer T., Peters C., Fugazzola L., Cassio A., Heinrichs C., Beauloye V., Pohlenz J. (2021). Congenital Hypothyroidism: A 2020–2021 Consensus Guidelines Update—An ENDO-European Reference Network Initiative Endorsed by the European Society for Pediatric Endocrinology and the European Society for Endocrinology. Thyroid.

[3] Nair P.S., Sobhakumar S., Kailas L. (2010). Diagnostic re-evaluation of children with congenital hypothyroidism. Indian Pediatr..

[4] Rabbiosi S., Vigone M.C., Cortinovis F., Zamproni I., Fugazzola L., Persani L., Corbetta C., Chiumello G., Weber G. (2013). Congenital Hypothyroidism With Eutopic Thyroid Gland: Analysis of Clinical and Biochemical Features at Diagnosis and after Re-Evaluation. J. Clin. Endocrinol. Metab..

[5] Cho M.S., Cho G.S., Park S.H., Jung M.H., Suh B.K., Koh D.G. (2014). Earlier re-evaluation may be possible in pediatric patients with eutopic congenital hypothyroidism requiring lower L-thyroxine doses. Ann. Pediatr. Endocrinol. Metab..

[6] Messina M.F., Aversa T., Salzano G., Zirilli G., Sferlazzas C., De Luca F., Lombardo F. (2015). Early Discrimination between Transient and Permanent Congenital Hypothyroidism in Children with Eutopic Gland. Horm. Res. Paediatr..

[7] Kara C., Günindi F., Yılmaz G.C., Aydın M. (2016). Transient Congenital Hypothyroidism in Turkey: An Analysis on Frequency and Natural Course. J. Clin. Res. Pediatr. Endocrinol..

[8] Kang M.J., Chung H.-R., Oh Y.-J., Shim Y.-S., Yang S., Hwang I.-T. (2017). Three-year follow-up of children with abnormal newborn screening results for congenital hypothyroidism. Pediatr. Neonatol..

[9] Park I.S., Yoon J.S., So C.H., Lee H.S., Hwang J.S. (2017). Predictors of transient congenital hypothyroidism in children with eutopic thyroid gland. Ann. Pediatr. Endocrinol. Metab..

[10] Saba C., Guilmin-Crepon S., Zénaty D., Martinerie L., Paulsen A., Simon D., Storey C., Dos Santos S., Haignere J., Mohamed D. (2018). Early Determinants of Thyroid Function Outcomes in Children with Congenital Hypothyroidism and a Normally Located Thyroid Gland: A Regional Cohort Study. Thyroid.

[11] Oron T., Lazar L., Ben-Yishai S., Tenenbaum A., Yackobovitch-Gavan M., Meyerovitch J., Phillip M., Lebenthal Y. (2018). Permanent vs. Transient Congenital Hypothyroidism: Assessment of Predictive Variables. J. Clin. Endocrinol. Metab..

[12] Higuchi S., Hasegawa Y. (2019). Levothyroxine dosages less than 2.4 μg/kg/day at 1 year and 1.3 μg/kg/day at 3 years of age may predict transient congenital hypothyroidism. Clin. Pediatr. Endocrinol..

[13] Park E.S., Yoon J.Y. (2019). Factors associated with permanent hypothyroidism in infants with congenital hypothyroidism. BMC Pediatr..

[14] Asena M., Demiral M., Unal E., Öcal M., Demirbilek H., Özbek M.N. (2020). Validity of Six Month L-Thyroxine Dose for Differentiation of Transient or Permanent Congenital Hypothyroidism. J. Clin. Res. Pediatr. Endocrinol..

[15] Long W., Zhou L., Wang Y., Liu J., Wang H., Yu B. (2020). Complicated Relationship between Genetic Mutations and Phenotypic Characteristics in Transient and Permanent Congenital Hypothyroidism: Analysis of Pooled Literature Data. Int. J. Endocrinol..

[16] Chen S.-H., Yang B.-C., Li J.-Y., Xu P., Wang F. (2021). Diagnostic re-evaluation and predictors of congenital hypothyroidism with eutopic thyroid gland in Jiangxi, China. J. Pediatr. Endocrinol. Metab..

[17] Nagasaki K., Sato H., Sasaki S., Nyuzuki H., Shibata N., Sawano K., Hiroshima S., Asami T. (2021). Re-Evaluation of the Prevalence of Permanent Congenital Hypothyroidism in Niigata, Japan: A Retrospective Study. Int. J. Neonatal Screen..

[18] Abbasi F., Janani L., Talebi M., Azizi H., Hagiri L., Rimaz S. (2021). Risk factors for transient and permanent congenital hypothyroidism: A population-based case-control study. Thyroid Res..

[19] Matejek N., Tittel S.R., Haberland H., Rohrer T., Busemann E.-M., Jorch N., Schwab K.-O., Wölfle J., Holl R.W., Bettendorf M. (2021). Predictors of transient congenital primary hypothyroidism: Data from the German registry for congenital hypothyroidism (AQUAPE “HypoDok”). Eur. J. Nucl. Med. Mol. Imaging.

[20] Mehran L., Azizi F., Mousapour P., Cheraghi L., Yarahmadi S., Amirshekari G., Khalili D. (2021). Development of a risk prediction model for early discrimination between permanent and transient congenital hypothyroidism. Endocrine.

[21] Yamamura H., Kokumai T., Furuya A., Suzuki S., Tanahashi Y., Azuma H. (2020). Increase in doses of levothyroxine at the age of 3 years and above is useful for distinguishing transient and permanent congenital hypothyroidism. Clin. Pediatr. Endocrinol..

[22] Kemper A.R., Grosse S.D., Baker M., Pollock A.J., Hinton C.F., Shapira S.K. (2020). Treatment Discontinuation within 3 Years of Levothyroxine Initiation among Children Diagnosed with Congenital Hypothyroidism. J. Pediatr..

[23] Itonaga T., Higuchi S., Shimura K., Nagasaki K., Satoh M., Takubo N., Takahashi I., Sawada H., Hasegawa Y. (2019). Levothyroxine Dosage as Predictor of Permanent and Transient Congenital Hypothyroidism: A Multicenter Retrospective Study in Japan. Horm. Res. Paediatr..

[24] Korzeniewski S.J., Grigorescu V., Kleyn M., Young W.I., Birbeck G., Todem D., Romero R., Paneth N. (2013). Transient Hypothyroidism at 3-Year Follow-Up among Cases of Congenital Hypothyroidism Detected by Newborn Screening. J. Pediatr..

[25] Hashemipour M., Hovsepian S., Kelishadi R., Iranpour R., Hadian R., Haghighi S., Gharapetian A., Talaei M., Amini M. (2009). Permanent and transient congenital hypothyroidism in Isfahan–Iran. J. Med. Screen..

[26] Unüvar T., Demir K., Abacı A., Ataş A., Büyükgebiz A., Böber E. (2013). Monitoring and prognostic evaluation of patients with congenital hypothyroidism treated in a pediatric endocrinology unit. Turk. J. Pediatr..

[27] Unüvar T., Demir K., Abacı A., Büyükgebiz A., Böber E. (2013). The Role of Initial Clinical and Laboratory Findings in Infants With Hyperthyrotropinemia to Predict Transient or Permanent Hypothyroidism. J. Clin. Res. Pediatr. Endocrinol..

[28] Aguiar L., Garb J., Reiter E., Visintainer P., Singh R., Allen H., Tonyushkina K. (2016). Can One Predict Resolution of Neonatal Hyperthyrotropinemia?. J. Pediatr..

[29] Razavi Z., Mohammadi L. (2016). Permanent and Transient Congenital Hypothyroidism in Hamadan West Province of Iran. Int. J. Endocrinol. Metab..

[30] Fu C., Luo S., Li Y., Li Q., Hu X., Li M., Zhang Y., Su J., Hu X., Chen Y. (2017). The incidence of congenital hypothyroidism (CH) in Guangxi, China and the predictors of permanent and transient CH. Endocr. Connect..

[31] Habib A., Shojazadeh A., Molayemat M., Habib A., Jeddi M., Arabsolghar R., Nahas M., Rahimi N., Ardekani F.M. (2021). Prevalence and predictive factors of transient and permanent congenital hypothyroidism in Fars province, Iran. BMC Pediatr..

[32] Zdraveska N., Zdravkovska M., Anastasovska V., Sukarova-Angelovska E., Kocova M. (2018). Diagnostic re-evaluation of congenital hypothyroidism in Macedonia: Predictors for transient or permanent hypothyroidism. Endocr. Connect..

